# Supplementary material for: Respiratory viruses Ct values and association with clinical outcomes among adults visiting the ED with lower respiratory tract infections
Source: PLoS One. 2025 May 29;20(5):e0320503. doi: 10.1371/journal.pone.0320503 (PMC12122020; doi:10.1371/journal.pone.0320503)
Supplement: S1 Table — (DOCX) [file pone.0320503.s001.docx]

| **Variable** | **E229** | **HKU1** | **Influenza A** | **Influenza B** | **Metapneumovirus** | **NL63** | **OC43** | **PIV** | **Rhino** | **RSV** | **SARS-CoV-2** | **p** | **q** |
| --- | --- | --- | --- | --- | --- | --- | --- | --- | --- | --- | --- | --- | --- |
| Male | 3 (75%) | 9 (69%) | 46 (53%) | 6 (54%) | 8 (35%) | 3 (60%) | 4 (57%) | 7 (54%) | 44 (53%) | 21 (53.8%) | 48 (62.3%) | 0.693 | 0.71 |
| Cough | 2 (50%) | 2 (16%) | 49 (60%) | 7 (64%) | 14 (64%) | 4 (80%) | 3 (43%) | 6 (46%) | 49 (65.3%) | 22 (59.5%) | 42 (59.2%) | 0.203 | 0.26 |
| Dyspnea | 4 (100%) | 5 (41%) | 58 (71%) | 6 (54%) | 15 (68%) | 3 (60%) | 3 (43%) | 7 (53%) | 57 (76%) | 30 (81.1%) | 54 (76.1%) | 0.083 | 0.13 |
| Chest pain | 0 (0%) | 2 (16%) | 8 (10%) | 3 (27%) | 4 (18%) | 1 (20%) | 0 (0%) | 2 (15%) | 9 (12%) | 7 (18.9%) | 6 (8.5%) | 0.546 | 0.58 |
| Rhinorrhea | 0 (0%) | 0 (0%) | 6 (7%) | 1 (9%) | 2 (9%) | 0 (0%) | 1 (14%) | 1 (8%) | 11 (14.7%) | 3 (8.1%) | 1 (1.4%) | 0.212 | 0.26 |
| Odynophagia | 0 (0%) | 2 (16%) | 1 (1%) | 0 (0%) | 0 (0%) | 0 (0%) | 0 (0%) | 0 (0%) | 0 (0%) | 2 (5.4%) | 2 (2.8%) | 0.129 | 0.19 |
| Myalgia | 1 (25%) | 0 (0%) | 18 (22%) | 3 (27%) | 1 (4%) | 0 (0%) | 0 (0%) | 0 (0%) | 3 (4.1%) | 3 (8.1%) | 20 (28.2%) | 0.001 | 0.001 |
| Headache | 0 (0%) | 0 (0%) | 7 (8%) | 1 (9%) | 0 (0%) | 1 (20%) | 1 (14%) | 0 (0%) | 4 (5.3%) | 2 (5.4%) | 11 (15.5%) | 0.264 | 0.312 |
| Abdominal pain | 0 (0%) | 5 (41%) | 7 (8%) | 2 (18%) | 2 (9%) | 0 (0%) | 1 (14%) | 1 (8%) | 5 (6.7%) | 0 (0%) | 14 (19.7%) | 0.004 | 0.010 |
| Asthenia | 1 (25%) | 3 (25%) | 28 (34%) | 3 (27%) | 8 (36%) | 1 (20%) | 4 (57%) | 3 (23%) | 13 (17.3%) | 8 (21.6%) | 34 (47.9%) | 0.013 | 0.029 |
| Malaise | 1 (25%) | 3 (25%) | 10 (12%) | 2 (18%) | 3 (14%) | 1 (20%) | 1 (14%) | 2 (15%) | 3 (4%) | 2 (5.4%) | 2 (2.9%) | 0.029 | 0.053 |
| Fever | 0 (0%) | 6 (50%) | 60 (73%) | 7 (63%) | 12 (54%) | 3 (60%) | 4 (57%) | 4 (31%) | 27 (36%) | 16 (43.2%) | 53 (74.6%) | 0.001 | 0.001 |
| High blood pressure | 2 (50%) | 6 (50%) | 41 (50%) | 5 (45%) | 14 (64%) | 3 (60%) | 4 (57%) | 9 (69%) | 41 (54.7%) | 21 (58.3%) | 35 (49.3%) | 0.947 | 0.947 |
| Diabetes | 1 (25%) | 6 (50%) | 20 (24%) | 1 (9%) | 5 (22.7%) | 2 (40%) | 1 (14%) | 4 (31%) | 15 (20%) | 12 (33.3%) | 20 (28.2%) | 0.512 | 0.562 |
| Obesity | 2 (50%) | 1 (8.3%) | 5 (6.3%) | 0 (0%) | 1 (4.8%) | 0 (0%) | 0 (0%) | 1 (8%) | 4 (5.3%) | 3 (8.3%) | 11 (15.5%) | 0.187 | 0.255 |
| Immunosuppression | 1 (25%) | 2 (16%) | 8 (9.8%) | 1 (9%) | 8 (36.4%) | 1 (20%) | 0 (0%) | 3 (23%) | 14 (18.7%) | 8 (22.2%) | 5 (7%) | 0.031 | 0.054 |
| Asthma | 0 (0%) | 2 (16%) | 11 (14%) | 0 (0%) | 0 (0%) | 0 (0%) | 1 (14%) | 1 (8%) | 9 (12.2%) | 2 (5.6%) | 4 (5.6%) | 0.024 | 0.046 |
| Fibrosis | 0 (0%) | 1 (8%) | 1 (1.2%) | 0 (0%) | 0 (0%) | 0 (0%) | 1 (14%) | 0 (0%) | 4 (5.4%) | 4 (11.1%) | 1 (1.4%) | 0.148 | 0.208 |
| BPCO | 1 (25%) | 1 (8%) | 17 (21%) | 0 (0%) | 1 (4.5%) | 0 (0%) | 1 (14%) | 2 (15%) | 16 (21.6%) | 6 (16.7%) | 2 (2.8%) | 0.018 | 0.035 |
| Chronic renal failure | 1 (25%) | 0 (0%) | 13 (16%) | 0 (0%) | 4 (18.2%) | 0 (0%) | 1 (14%) | 4 (31%) | 6 (8%) | 9 (25%) | 9 (12.7%) | 0.132 | 0.192 |
| Chest CT | 0 (0%) | 3 (25%) | 8 (9%) | 1 (9%) | 4 (18.2%) | 0 (0%) | 1 (14%) | 1 (8%) | 16 (21.3%) | 5 (13.5%) | 63 (87.5%) | 0.001 | 0.001 |
| CT finding: Pneumonia | 1 (25%) | 3 (25%) | 17 (21%) | 2 (20%) | 10 (45.5%) | 3 (60%) | 4 (57%) | 3 (23%) | 18 (24%) | 7 (18.9%) | 65 (90.3%) | 0.001 | 0.001 |
| Antibiotics | 2 (50%) | 6 (50%) | 48 (59%) | 4 (40%) | 16 (72.7%) | 3 (60%) | 5 (71%) | 5 (38%) | 36 (48%) | 19 (51.4%) | 46 (64.8%) | 0.34 | 0.389 |
| Hospitalization | 3 (75%) | 9 (75%) | 64 (79%) | 6 (54%) | 18 (81.8%) | 4 (80%) | 6 (86%) | 13 (100%) | 58 (77.3%) | 30 (81.1%) | 62 (88.6%) | 0.210 | 0.265 |
| Medical ward | 3 (75%) | 9 (75%) | 60 (74%) | 6 (54%) | 18 (81.8%) | 4 (80%) | 6 (86%) | 13 (100%) | 57 (76%) | 29 (78.4%) | 60 (87%) | 0.228 | 0.278 |
| ICU | 1 (25%) | 0 (0%) | 19 (24%) | 0 (0%) | 1 (5.3%) | 0 (0%) | 2 (29%) | 3 (30%) | 16 (23.2%) | 2 (6.2%) | 21 (32.3%) | 0.016 | 0.034 |
| Mechanical ventilation | 0 (0%) | 0 (0%) | 7 (9%) | 0 (0%) | 1 (5.3%) | 0 (0%) | 0 (0%) | 0 (0%) | 7 (10.4%) | 1 (3%) | 10 (16.1%) | 0.682 | 0.709 |
| Hospital death | 0 (0%) | 2 (16.7%) | 4 (5%) | 0 (0%) | 2 (9.5%) | 1 (20%) | 1 (14%) | 5 (38%) | 8 (10.8%) | 5 (13.9%) | 12 (17.6%) | 0.046 | 0.077 |
| Deaths D28 | 0 (0%) | 2 (16.7%) | 4 (5%) | 1 (9%) | 2 (9.5%) | 1 (20%) | 1 (14%) | 5 (38%) | 8 (11%) | 5 (13.9%) | 12 (17.6%) | 0.080 | 0.128 |
| Medium Ct | 2 (50%) | 7 (53.8%) | 47 (55%) | 6 (54%) | 11 (47.8%) | 1 (20%) | 5 (71%) | 3 (23%) | 50 (60.2%) | 11 (28.2%) | 36 (46.8%) | 0.001 | 0.001 |
| High Ct | 2 (50%) | 4 (30.8%) | 23 (27%) | 0 (0%) | 4 (17.4%) | 4 (80%) | 2 (29%) | 8 (61%) | 24 (28.9%) | 10 (25.6%) | 28 (36.4%) |  |  |
| Low Ct | 0 (0%) | 2 (15.4%) | 16 (19%) | 5 (45%) | 8 (34.8%) | 0 (0%) | 0 (0%) | 2 (15%) | 9 (10.8%) | 18 (46.2%) | 13 (16.9%) |  |  |

*Supplementary Table: Symptoms, Clinical patterns and outcomes according to the pathogen*

*Statistic: Fisher exact tests*
