# Supplementary material for: Respiratory viruses Ct values and association with clinical outcomes among adults visiting the ED with lower respiratory tract infections
Source: PLoS One. 2025 May 29;20(5):e0320503. doi: 10.1371/journal.pone.0320503 (PMC12122020; doi:10.1371/journal.pone.0320503)
Supplement: Attachment — Submitted filename: PONE-D-24-46715.pdf [file pone.0320503.s002.pdf]

Manuscript Number: PONE-D-24-46715

Title: Respiratory viruses Ct values and association with clinical outcomes among adults visiting the ED with lower respiratory tract infections.

Overview and general recommendation:

This subject is important approximately because this study evaluates the association between Ct values and clinical outcomes in patients tested via point-of care testing upon emergency department (ED) admission.

I found the manuscript well written approximately, well described approximately.

I listed **minor points** below for editing.

Comments to Authors:

Minor points

1- Rewrite the title of manuscript; do not use the abbreviations in the title.

2- In abstract, page2, 35line\ In methods, page5, 108,119&147line\ In results, page9, 173line\ In discussion,page13,219line, 224line, 226line, 234line and 255line\ In conclusion, page14, 260line; you wrote (11 times We)! The rule of manuscript writing is to avoid using (We). So you should delete (We) and use academic scientific words such as (This study or The current study or The present study).

3- In results; you wrote the title of all tables below the tables!!! That's incorrect. Kindly transfer the table titles' above the tables in addition to that you should write the meaning of each abbreviation inside the tables below each table.

Kind regards
